# Outcomes of Young Patients With Alcoholic Cirrhosis After First Hospitalization for Cirrhosis: A Carilion Clinic Experience

**DOI:** 10.7759/cureus.16695

**Published:** 2021-07-28

**Authors:** Shravani R Reddy, Mohamad Mouchli, Robert Summey, Chirstopher Walsh, Adil Mir, Lindsey Bierle, Marrieth G Rubio

**Affiliations:** 1 Gastroenterology, Virginia Tech Carilion School of Medicine, Roanoke, USA; 2 Gastroenterology, Cleveland Clinic, Cleveland, USA; 3 Medicine, University of Pennsylvania, Philadelphia, USA; 4 Internal Medicine, Carilion Clinic, Roanoke , USA; 5 Internal Medicine, Carilion Clinic, Ronaoke , USA; 6 Internal Medicine, Carilion Clinic, Roanoke, USA; 7 Gastroenterology and Hepatology, Virginia Tech Carilion School of Medicine, Roanoke, USA

**Keywords:** alcoholic liver disease, young cirrhosis, alcoholic cirrhosis

## Abstract

Background

Alcoholic cirrhosis though uncommon in young patients is being reported more frequently and related mortality is also increasing.

Study aim

To evaluate risk factors associated with mortality among young patients (<40 years) with alcoholic cirrhosis and older patients (> 40 years old) after their first hospitalization in a tertiary referral academic center.

Methods

Carilion clinic’s electronic medical record (EPIC) was queried to identify all alcoholic patients hospitalized for the first time with either a new diagnosis of alcoholic cirrhosis or a prior diagnosis of this from 2008 to 2016 with follow-up through June 2018. Information on demographics, comorbidities, lab values, procedures, and mortality was extracted. The cumulative risks of long-term mortality after the first hospitalization were estimated using Kaplan-Meier curves and compared between the two groups; those < 40 years of age and those > 40 years of age. Demographic data, lab values, and comorbidities associated with cirrhosis were assessed using multivariable Cox proportional hazard analysis to determine risk factors associated with long-term mortality.

Results

We identified 65 young patients out of a total of 325 patients admitted for the first time for alcoholic cirrhosis (mean age: 34.6 ± 4.7 yrs, 72.3% males, 74.4% current alcohol users, 52.3% current smokers, 12.6% current illicit drugs users). The one, three, and five-year cumulative mortality after the first hospitalization was 21.1 %, 31.1%, and 49.7% respectively. The median survival for young patients was longer as compared to the older patients (p<0.001); likely related to high early mortality in older patients who had many other comorbidities. On multivariate Cox proportional hazard analysis, increased age [hazard ratio (HR) 1.03; 95% confidence interval (CI), 1.01-1.05], neutrophils-to-lymphocytes ratio (NLR) at first hospital discharge (HR 1.02; 95% CI, 1.01-1.04), the presence of encephalopathy (HR, 1.93; 95% CI, 1.06-3.55), and initial MELD (model for end-stage liver disease) score (HR, 1.13; 95% CI, 1.08-1.19) were associated with increased risk of mortality. Though the majority of patients endorsed current alcohol and tobacco use before the admission, it was not significantly associated with mortality.

Conclusions

Five-year cumulative mortality for patients < 40 years of age with alcoholic cirrhosis after their first hospitalization is 49.7%. Old age, most recent NLR, hepatic encephalopathy, and MELD score on admission were associated with increased late mortality.

## Introduction

Cirrhosis or chronic liver disease due to alcohol is associated with frequent hospitalization and fatal complications which create socioeconomic burdens on patients, families, and the healthcare system [1]. The World Health Organization (WHO) implemented a strategy to reduce the harmful use of alcohol since alcohol misuse accounts for about 6% of worldwide mortality [2]. Tapper et al. also reported that the annual mortality due to alcohol-related cirrhosis and alcohol use disorder has increased dramatically in many states over the past few years [3]. Another study showed that the age-standardized mortality rate from alcohol use disorders increased nationally by 3.2% between 2000 and 2014 [4]. In another study between 1979-2008, patients younger than 45 years of age with alcoholic liver cirrhosis, chronic hepatitis, and cirrhosis were noted to have a younger age at death [5]. In the state of Virginia, alcohol-related cirrhosis mortality increased between 1999-2016 from about 8% to 12% [3]. 

The increasing incidence of young patients with alcoholic liver disease represents one of the largest burdens on the health care system in the near future [3, 4]. In an observational cohort study by Tapper et al. during the years 1999-2016 people aged 25-34 years with alcoholic cirrhosis experienced the highest average annual increase in cirrhosis-related mortality [3]. The most significant factors contributing to mortality were sepsis and spontaneous bacterial peritonitis [3]. 

Clinical observations from Carilion Clinic Roanoke Memorial Hospital have identified the increasing mortality of young patients with alcoholic cirrhosis and prompted us to suspect that there are under-appreciated factors contributing to these patients’ mortality. A better understanding of factors associated with mortality may help clarify targets for improvement in care and subsequently help decrease healthcare utilization. 

## Materials and methods

Study population 

This is an IRB-approved retrospective cohort study of patients with alcoholic cirrhosis who were treated during their first hospital admission for liver disease at Carilion Roanoke Memorial Hospital (CRMH) in Roanoke, Virginia between August 1, 2008, until November 30, 2016, with follow-up through June 30, 2018. We included all identified patients ≥ 18 years of age diagnosed with alcoholic liver cirrhosis (identified by hospital diagnosis ICD 10 code 70.3) and collected data regarding demographics (age and sex), illicit drug use, lab values [hemoglobin and platelet counts, liver function tests, the neutrophil-lymphocyte ratio (NLR) and MELD score], and history of complications of end-stage liver disease (ESLD) including portal hypertensive bleeding, ascites, spontaneous bacterial peritonitis (SBP) and hepatic encephalopathy (HE). Additional chart review was performed using the medical record numbers (MRNs) to determine biopsy status and evaluation for transplantation. Current admission data was used as the beginning date of the study. Late survival was defined as survival post-discharge up to five years. We identified 65 patients with alcoholic liver cirrhosis ages 20 to 40 and matched them to 260 patients (at a 1:4 ratio) with alcoholic cirrhosis patients older than 40 years old, hospitalized over the same time period, and compared survival. 

Statistical analysis 

The data were reported as mean (± SD), median (interquartile range, IQR), ranges, and categorical variables by counts and percentages as appropriate. Estimates of the mortality rates were determined by using the Kaplan-Meier survival curve with a log-rank test. To identify risk factors associated with early mortality, we performed a univariate time-to-event analysis with Cox proportional regression models that accounted for the case-cohort design by using case weights to account for the sampling frame and robust estimates of variance. Variables with p < 0.05 on univariate analysis were included in a multivariable Cox proportional hazard analysis used to identify independent risk factors associated with mortality at five years. All statistical analyses were conducted using JMP version 10 for Windows (SAS Institute Inc., Cary, NC, United States). 

## Results

Patient eligibility and demographics 

Sixty-five young patients (age < 40 years old) with alcoholic cirrhosis were identified. These 65 patients were compared to 260 randomly selected controls (alcoholic cirrhosis patients older than 40 years old). The mean age at the time of admission was 34.6 ± 4.7 years for the young group and 58.2 ± 9.4 years for the control group. 

There were 72.3% men in the young group and 72.9% men in the control group. Seven young patients (10.8%) had biopsy-proven alcoholic cirrhosis the others were diagnosed clinically. Active alcohol use before admission was noted in 55 young patients (84.6%) and 187 control patients (72.2%). There were 14 young patients (21.5%, p = 0.01) and 27 control patients (19.4%, p = 0.01) who endorsed active illicit drug use. Other findings are shown in Table 1. 

**Table 1:** Comparison of clinical and demographic characteristics between cases who were hospitalized for alcoholic cirrhosis at an age younger than 40 years old and controls who are older than 40 years old AST: aspartate transaminase, ALT: alanine transaminase

Characteristics n (%)	<40 ( n=65)	>40 ( n=260)	P-value
Male gender	47 (72.3%)	200 (76.9%)	0.441
Age on admission (mean ± SD)	34.6±4.7	58.2±9.4	<0.01
Illicit drug use	14 (21.5%)	27 (19.42%)	<0.01
Alcohol Use	55 (84.6 %)	187 (72.2%)	0.045
Tobacco use	43 (66.2%)	127 (49.0%)	0.694
ALT at discharge (mean ± SD)	60.0±61.3	85.8±205.4	<0.01
AST at discharge (mean ± SD)	135.0±187.9	200.5±582.1	<0.01
Platelet counts at discharge ( mean ± SD)	133.7±103.9	153.6±113.2	<0.01
Hepatic Encephalopathy	37 (56.9%)	147 (56.5%)	0.994
Ascites	47 (72.3%)	159 (61.2%)	0.097
SBP	11 (16.9%)	36 (13.8%)	0.503
varices	39 (60.0%)	78 (30.0%)	<0.01
Liver transplantation	5 (7.7%)	9 (3.5%)	0.271
Neutrophils to Lymphocytes Ratio at discharge(NLR) (mean ± SD)	5.1±5.3	7.2±8.6	<0.01
MELD on admission (mean ± SD)	10.4±5.0	10.4±5.3	0.965

Risk factors associated with mortality 

By univariate analysis, patient’s age (HR 1.02; 95% CI, 1.01-1.04, P < .01); MELD score on admission (HR 1.14; 95% CI, 1.10-1.17, P < .01); ALT levels at discharge (HR 1.002; 95% CI, 1.001-1.003, P < .01), aspartate transaminase (AST) levels at discharge; (HR 1.0007; 95% CI, 1.0004-1.001, P < .01) platelet counts at discharge (HR 0.998; 95% CI, 0.996-0.999, P < .01); NLR at discharge (HR 1.03; 95% CI, 1.01-1.04, P < .01); the presence of ascites (Yes vs No) (HR 1.76; 95% CI, 1.21-2.55, P <0.01); the presence of spontaneous bacterial peritonitis (Yes vs No) (HR 2.28; 95% CI, 1.57-3.31, P <0.01); and the presence of hepatic encephalopathy (Yes vs No) (HR 2.24; 95% CI, 1.57-3.21, P <0.01) were all significant factors associated with increased mortality. In a multivariable model that included the patient’s age, NLR at first hospital discharge, alanine transaminase (ALT) levels at first hospital discharge, AST levels at first hospital discharge, platelet counts at first hospital discharge, the presence of hepatic encephalopathy on admission, and initial MELD score, age on admission (HR 1.03; 95% CI, 1.01-1.05), NLR at discharge (HR 1.02; 95% CI, 1.01-1.04), hepatic encephalopathy (Yes vs No) (HR 1.93; 95% CI, 1.06-3.55), and MELD score on admission (HR 1.13; 95% CI, 1.08-1.19) were all associated with increased risk of mortality (Table 2). Though alcohol and tobacco use before admission was endorsed by a majority of patients neither was identified as a risk factor for decreased long-term mortality in either group. 

**Table 2:** Risk Factors for long term overall mortality of hospitalized patients with alcoholic cirrhosis

Source	Hazard Ratio	P-Value	Hazard Ratio	P-Value
Age (per year)	1.02 (1.01-1.04)	<0.01	1.03 (1.01-1.05)	<0.01
Sex (M:F)	0.94 (0.66-1.34)	0.734	-	-
Illicit drug use (Y: N)	1.19 (0.76-1.88)	0.440	-	-
Alcohol Use (Y: N)	0.995 (0.69-1.44)	0.978	-	-
Tobacco use (Ever: Never)	0.78 (0.55-1.12)	0.180		-
ALT at discharge	1.002 (1.001-1.003)	<0.01	1.0005 (0.999-1.002)	0.589
AST at discharge	1.0007 (1.0004-1.001)	<0.01	1.0002 (0.9997-1.0008)	0.333
Platelet counts at discharge	0.998 (0.996-0.999)	<0.01	0.998 (0.996-1.0007)	0.170
Number of hospitalizations	0.95 (0.86-1.02)	0.141	-	-
Hepatic Encephalopathy (Y: N)	2.24 (1.57-3.21)	<0.01	2.0 (1.09-3.67)	0.032
Ascites (Y: N)	1.76 (1.21-2.55)	<0.01	1.26 (0.69-2.30)	0.526
SBP (Y: N)	2.28 (1.57-3.31)	<0.01	1.41 (0.78-2.54)	0.841
Esophageal varices (Y: N)	1.12 (0.81-1.53)	0.499	-	-
Neutrophils to Lymphocytes Ratio (NLR) at discharge	1.03 (1.01-1.04)	<0.01	1.020 (1.01-1.04)	0.028
MELD on admission	1.14 (1.10-1.17)	<0.01	1.13 (1.08-1.19)	<0.01

Long-term survival between young patients and controls 

**Figure 1:**
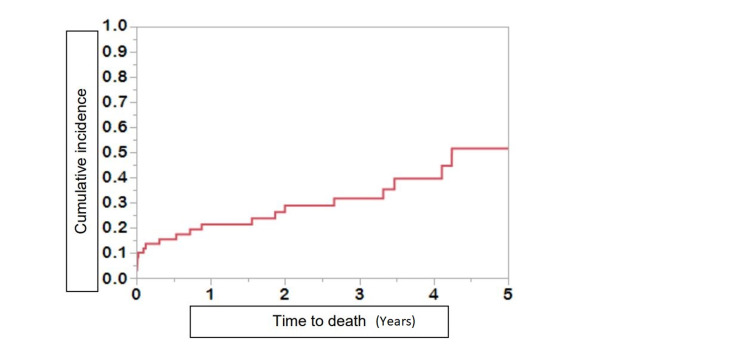
Cumulative incidence of death after first hospitalization for young patients with alcoholic cirrhosis

**Figure 2:**
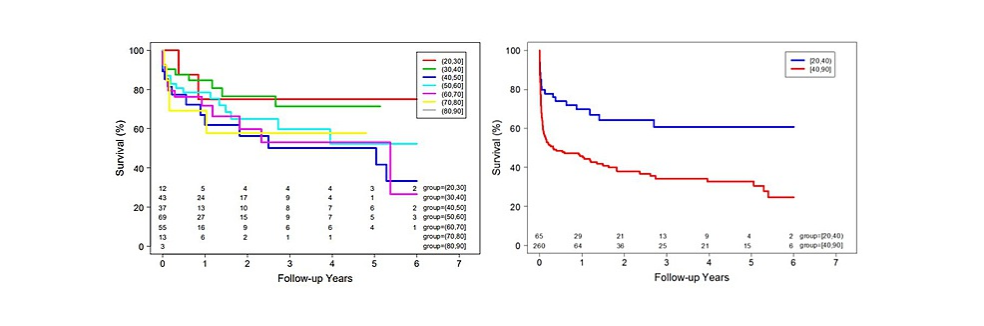
Late survival after first hospitalization Figure 2A: Kaplan-Meier Curves for mortality among hospitalized patients per age groups showed that patient survival decreases for elder age groups. Figure 2B: Kaplan-Meier Curves for mortality among hospitalized alcoholic cirrhosis patients <40 years old (Group A) and > 40 years old (Group B) indicates that group B experienced higher late mortality compared to those in group A.

**Figure 3:**
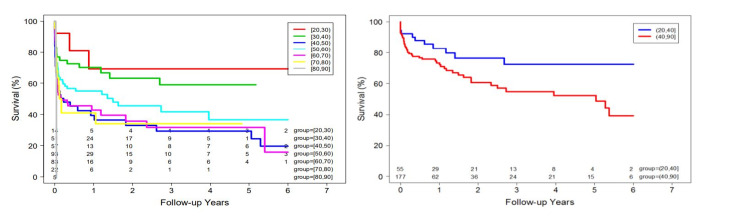
Overall survival after first hospitalization Figure 3A: Kaplan-Meier Curves for mortality among hospitalized patients per age groups showed that patient survival decreases for elder age groups. Figure 3B: Kaplan-Meier Curves for mortality among hospitalized alcoholic cirrhosis patients <40 years old (Group A) and > 40 years old (Group B) indicates that group B experienced higher late mortality compared to those in group A.

We reviewed data over up to a five-year period. The mean follow-up after the first hospitalization was 1.15 years ± 1.9 for cases and 2.0 years ± 1.9 for controls. At last follow-up, twenty cases (30.8%) and 135 controls (52%) had died. Eleven deceased young patients (55.0%) and thirty-nine deceased control patients (28.9%) denied alcohol use prior to admission. In both groups, complications from cirrhosis were the most common cause of reduced survival and death. The cumulative incidence for overall mortality in young alcoholic cirrhosis patients after the first hospitalization was 21.1 %, 31.1%, and 49.7% at one, three, and five years, respectively (Figure 1). Despite liver transplantation in 14 patients, 2/5 young patients and 6/9 controls died. Older patients diagnosed with alcoholic cirrhosis experienced higher overall and long-term mortality compared to young patients with alcoholic cirrhosis (P<0.01) (Figure 2, 3). 

## Discussion

We found that older age, hepatic encephalopathy, NLR at discharge, and baseline MELD score were associated risk factors that predicted poor outcomes in these patients. Despite 84.6% of these patients endorsing active alcohol use before admission, alcohol cessation was not a defining factor for improved survival. This study shows that the overall mortality for patients < 40 years old with alcoholic liver cirrhosis hospitalized in Southwest Virginia is unexpectedly high. This high mortality is still lower than the mortality observed in older patients (> 40 years old) and the most common cause of mortality was complications of cirrhosis for both groups which included ascites, spontaneous bacterial peritonitis, and hepatic encephalopathy.

It is commonly believed that end-stage liver disease secondary to alcohol rarely occurs in young adults as compared to older patients. This may be related to a lack of enrollment of young alcoholic patients in previous studies, though some studies reported the mortality to be very low in young patients but significantly high in the middle-age group [6, 7, 8, 9]. The Centers for Disease Control (CDC) and multiple recent studies demonstrate a spike in the incidence, prevalence, and mortality of alcoholic cirrhosis in young patients over the last decade [10]. 

In our study, we confirmed the previous findings by Rehm et al., who described mortality secondary to alcoholic cirrhosis as lower among patients younger than the age of 35 when compared to older patients before 2010 [11]. Another study reported that death rates secondary to chronic liver disease and cirrhosis from all causes are higher in patients > 45 years old compared to younger patients [12]. Although Tapper et al. and a national surveillance report by The National Institute on Alcohol Abuse and Alcoholism reported a significant increase in mortality among people aged 25-34 years over the last decade especially in white males; the mortality is still lower than older patients with other comorbidities such as coronary artery disease [3]. This is consistent with the fact that deaths due to alcoholic cirrhosis did not increase rapidly in the state of Virginia per the same study [3]. 

Alcohol intake has been recognized as a significant prognosis factor in liver cirrhosis [13]. In our study, we found that a majority (84.6%) of young patients endorsed active alcohol use on initial hospitalization but this factor was not significantly associated with a reduction in long-term mortality post-hospitalization. Verrill et al. also reported that alcohol abstinence is a key factor in improving prognosis even in patients with severe degrees of cirrhosis on biopsy [14]. Leon et al. also reported that a substantial decrease in alcohol consumption resulted in a marked immediate decrease in liver cirrhosis mortality [15]. Data about recurrent alcohol drinking after hospital discharge and duration of cessation before admission was not available to us and this likely affected our results. 

Several factors have been studied in the past and were found to be associated with increased mortality in patients with alcoholic cirrhosis [16-19]. We confirmed Alvarez et al. findings that survival secondary to alcoholic cirrhosis is highly associated with baseline MELD score, increasing age, and hepatic encephalopathy development [16]. Though we did not find ongoing alcohol use at the time of admission to be a risk factor for mortality; we found high NLR at hospital discharge to be a risk factor for late mortality. To our knowledge, this factor was not studied in the past as a risk factor for poor survival in patients with alcoholic cirrhosis, and further studies are warranted to confirm the association. 

Hazardous alcohol consumption and alcoholic cirrhosis are associated with large social, economic, and disease burdens. Although a study showed that alcohol control laws did not have a positive impact on hazardous alcohol consumption in some countries [1], and the incidence of excessive alcohol use in the young seems to be increasing, we still believe that other interventions should be undertaken as a population-wide priority to reduce alcohol consumption, such as education regarding alcohol risks and the benefits of moderation in contrast of total abstinence which may have a better impact in behavior. 

The retrospective nature of this study and the small sample size is certainly a limitation, as well as the fact that we were not able to collect data on patients who were admitted previously to other facilities due to logistic reasons. We were also not able to collect data on some variables such as the length of abstinence from alcohol, the patient’s lack of reliability regarding their drinking habits, and drinking status after discharge. These factors could significantly affect any conclusions regarding the effect of alcohol abstinence on survival. Another limitation is that we only studied patients undergoing hospital admissions and excluded patients with alcoholic liver cirrhosis who follow-up in the clinic and had never been hospitalized for decompensation. 

## Conclusions

In conclusion, our data demonstrate that there is a rising trend in mortality in young hospitalized patients with alcoholic liver cirrhosis but it is still lower than that of older patients (Age > 40 years old). Development of cirrhosis complications such as hepatic encephalopathy, high baseline MELD score, increasing age, and high NLR at discharge were factors associated with the increased mortality risk. 
